# The relationship between father absence and hostility among Chinese depressed youths: A serial mediation model and the role of self-esteem and frustration tolerance

**DOI:** 10.3389/fped.2022.711241

**Published:** 2023-01-26

**Authors:** Xiao-Ge Liu, Yang Li, Fang Xiong, Wen-Tian Li, Lian-Zhong Liu, Sullivan John S.

**Affiliations:** ^1^ Research Center for Psychological Sciences and Health, China University of Geosciences, Wuhan, China; ^2^ Department of Student Affairs Management, Zhejiang University of Water Resources and Electric Power, Hangzhou, China; ^3^ Moral Education and Art Department of Henan Vocational College of Geology and Mineral Resources, Zhengzhou, China; ^4^ Department of Clinical Psychology, Affiliated Wuhan Mental Health Center, Tongji Medical College of Huazhong University of Science & Technology, Wuhan, China; ^5^ Central Clinical School, University of Sydney, Sydney, NSW, Australia

**Keywords:** major depressive disorder, father absence, self-esteem, frustration tolerance, hostility

## Abstract

**Background:**

While the association between father absence and youth hostility has been well-documented among depressed youths, there is a lack of research on the potential mechanism underlying such an association. This study aimed to test a serial mediation model of self-esteem and frustration tolerance on the link between father absence and youth hostility.

**Methods:**

A total of 137 Chinese youths with major depressive disorder were recruited from Wuhan Mental Health Center. They completed a survey including the Father Absence Questionnaire to measure father absence, the Chinese Hostility Inventory (CHI) to measure hostility, the Psychological Endurance Questionnaire to measure frustration tolerance, and the Self-esteem Scale (SES) to measure self-esteem. A series of multiple linear regression models were employed to assess the associations among father absence, self-esteem, frustration tolerance, and hostility.

**Results:**

Although father absence was modestly associated with hostility (*r* = 0.30, *p *< 0.001), subsequent serial mediation analysis showed that father absence was not directly related to hostility (*β *= 0.06, *p *= 0.29) when self-esteem and frustration tolerance were included in the model. High levels of father absence had an adverse effect on levels of self-esteem, which decreased levels of frustration tolerance, and thus higher levels of hostility among depressed youths. The indirect effects of father absence on hostility through self-esteem, frustration tolerance, as well as through self-esteem and frustration tolerance serially accounted for 28%, 24%, and 24% of the total effect, respectively.

**Conclusion:**

Our study tested a serial mediation model of self-esteem and frustration tolerance as mediators between father absence and hostility among depressed youths. The findings strengthened our understanding of the potential mechanism underlying the association between self-esteem and frustration tolerance, which may provide useful guidance for future intervention programs.

## Introduction

Globally, major depressive disorder affects over 300 million people and is estimated to be the second leading cause of disability-adjusted life years (1, 2). Major depressive disorder (MDD) has an early onset that usually begins in childhood or young adulthood and is associated with a high risk of suicide (1, 3). The youth are at high risk for developing major depressive disorder, with an estimated prevalence ranging from 8% to 20% before 18 (4–6). According to the World Health Organization, major depressive disorder is the second most common cause of death among people aged 15–29 (1). Major depressive disorder in young people not only negatively affects the individuals themselves, but also causes a significant social and economic burden to the individual's family and to society (7, 8).

The close link between major depressive disorder and hostility has been widely reported in many studies, with hostility playing an essential role in both the onset and the sustainment of major depressive disorder (9–12). Hostility refers to negative beliefs and affection towards others and is characterized by negative attitudes such as suspicion, cynicism, and mistrust (13). Hostility is associated with both externalizing behaviors such as bullying and aggression, and internalizing affect disorders such as anxiety and depression (14). Previous research has consistently shown an elevated risk for hostility in depressed youths, with these individuals showing a much higher prevalence and level of hostility than non-depressed youths, as reported by both the individuals themselves and their parents (15). In fact, the most recent version of the Diagnostic and statistical manual of mental disorders (5th ed.) has listed the sustained presence of hostility as part of the diagnostic criteria for major depressive disorder in children and adolescents (16).

Understanding the causes and mechanism of hostility among depressed youths carries significant clinical implications in guiding future prevention and intervention strategies to promote better recovery. Among various risk factors reported in the literature, lack of parental care has been listed as an important contributor to hostility in the youths (17–19). Karen Horney (20) mentioned in her book “The Neurotic Personality of Our Time” that children will feel insecure if they do not receive or lack genuine care from their parents in childhood, thus triggering hostility. Father absence, the most common type of parental care lacking in the male-dominant world, plays an important role in youth hostility. Father absence is a broad term that encompasses a wide range of circumstances, which can be generally classified into physical absence (such as non-existence in one's life, death, divorce, absence for work commitments, incarceration, or institutionalization), and functional absence (such as absence due to disinterest or neglect despite physical presence) (21). An overwhelming majority of studies have documented the important and positive role a father's presence played in promoting youths' physical, psychological, and social development and adjustment (21, 22). Such positive impacts are even independent of the mother's presence and other social and familial factors (23). While the causal association between father absence and hostility has been well-documented, there is a lack of research on the potential mechanism underlying such an association in young people.

Two potential pathways between father absence and hostility in young people are issues around self-esteem and frustration tolerance. Self-esteem is defined as the “individual”s subjective evaluation of her or his worth as a person” (24). Abundant evidence has shown father absence has a significant impact on self-esteem (25–28). One study reported that youths brought up in single families, especially those where the father was absent, showed significantly lower levels of self-esteem than adolescents brought up in two-parent families (25). Lower self-esteem has been documented to be associated with a range of adverse health outcomes among young people, including depression, hostility, and violence. Longitudinal studies have demonstrated low self-esteem as an important contributing risk factor predicting later hostility and violent behaviors (29, 30).

Frustration tolerance is defined as “an effective response to blocked-goal attainment” (31) and is characterized by “a negative emotional response triggered after the omission and/or devaluation of an expected reward” (32). Low frustration tolerance is an irrational belief that aversive situations cannot be adapted to or tolerated (33). Studies showed that individuals with an absent father have low frustration tolerance, and those with low frustration tolerance are at high risk of experiencing hostility (34, 35). In Martin and Dahlen's study on the relationship of irrational beliefs and anger, they found low frustration tolerance triggered anger expression, with a moderate effect size (34). In addition, low self-esteem has been shown to affect low frustration tolerance and one study showed that self-esteem levels influenced coping styles through frustration tolerance (36).

Previous evidence suggested that self-esteem and frustration tolerance may mediate the relationship between father absence and hostility. So far, however, no study has examined these potential mediators empirically. The present study used a clinical sample diagnosed with major depressive disorder to assess the relationship between father absence and hostility. We assessed self-esteem and frustration tolerance as mediators of this relationship and hypothesized that the relationship of father absence and hostility would be associated with self-esteem and frustration tolerance.

## Materials and methods

### Participants

The study was a cross-sectional survey conducted from September 2020 to February 2021 in Affiliated Wuhan Mental Health Center, Tongji Medical College of Huazhong University of Science and Technology. The center is Hubei's largest public mental health care provider and has 950 psychiatric beds and provides around 300,000 outpatient consultations annually. The depression ward is a department of this hospital, which mainly treats patients with major depressive disorder and bipolar disorder, with 60 authorized beds. A total of 137 eligible patients with major depressive disorder were enrolled from the depression department.

The inclusion criteria were as follows: (1) diagnosis of Major Depressive Disorder according to International Classification of Diseases, 10th revision, based on a review of medical records and a clinical interview; (2) aged 12–25 years old (the age boundary standard refers to previous research (37) and the characteristics of the development of youths’ self-concepts (38)); (3) able to communicate and understand the survey instructions; (4) willing to provide written informed consent.

The exclusion criteria were as follows: (1) coexisting developmental delay, or other severe organic disorders; (2) drug or alcohol abuse; (3) comorbid schizophrenia; (4) difficulty in communication due to severe physical or mental diseases.

We distributed the questionnaires to patients who met the above criteria and written informed consent forms were signed by all participants and their guardians (when necessary). Potential participants were made aware that participation was entirely voluntary and that they could refuse to take part without any penalty. Participants were reassured anonymity and confidentiality. The study protocol was approved by the Ethics Committee of Wuhan Mental Health Center.

## Measures

### Father absence

Father absence was measured by the Father Absence Questionnaire developed by Zhu (39). The questionnaire is a widely used domestic scale for father absence assessment in China and includes two items: physical absence and functional absence. The total score ranges from 2 to 7, with higher scores implying a higher level of father absence.

### Hostility

Hostility was measured by the Chinese Hostility Inventory (CHI) compiled by Lin (40), which was adapted from Spielberger's State-Trait anger expression inventory and anger expression scale (41, 42). The CHI is a 20-item scale under four domains: hostile cognition, hostile emotion, expression of hostility, and suppression of hostility. Each item is scored on a 5-point Likert scale from 1 (strongly agree) to 5 (strongly disagree). The total score ranges from 20 to 100 points, with a higher score indicating a higher hostility degree. The Cronbach's alpha of the CHI was 0.89 in the present study, indicating well-qualified internal consistency.

### Frustration tolerance

Frustration tolerance was assessed by the Psychological Frustration Tolerance Questionnaire compiled by Xie, Wu, and Qin (43). The questionnaire includes 30 “yes-no” questions to assess youths' tolerance to various frustrating situations. Each item with a “yes” answer is counted as 1 point, and “no” is counted as 0 point. 12 negatively expressed items are reversely scored. The total score ranges from 0 to 30, with higher scores indicating higher levels of frustration tolerance. The Cronbach's alpha of the Questionnaire was 0.86 in the present study, which demonstrated well-qualified internal reliability in our sample.

### Self-esteem

Self-esteem was assessed by the Rosenberg Self-Esteem Scale (SES) (1965). The SES is a unidimensional questionnaire consisting of 10 items, in which items 3, 5, 8, 9, and 10 are reversely scored. Each item is scored on a 4-point Likert-type scale from 1 (strongly disagree) to 4 (strongly agree). The total score ranges from 10 to 40, with higher scores reflecting higher levels of self-esteem. The Cronbach's alpha of the SES was 0.85 in the present study.

### Procedure

The survey was conducted in Wuhan Mental Health Center. A total of 137 individuals with major depressive disorder completed an anonymous survey, which took approximately 40 min to complete. Three researchers reviewed the questionnaire for the study. After getting approval from the director and ward nurse, researchers visited each ward to approach each eligible patient and explained the purpose and process of the study to the patients and their families. After providing written informed consent, each eligible patient was invited to complete a questionnaire with the researchers’ assistance when necessary.

### Statistical analysis

SPSS 21.0 was used for data cleaning, coding, and preliminary analysis. All continuous variables were standardized. We used Spearman correlations to evaluate the associations among variables. Mediation analyses were carried out using SPSS PROCESS v.3.2 macro (44). A serial mediation model was proposed with self-esteem and frustration tolerance as first and second-order mediators in the association between father absence and hostility. In this study, we controlled for gender and age and bootstrapped 5,000 samples from the data. A 95% bootstrap confidence interval (CI) that did not include zero was considered significant.

## Results

### Participant characteristics

Participants consisted of 137 youths, including 56 (40.88%) males and 81 (59.12%) females. The mean age was 18.41 years (SD:3.69 years). Of the total sample, 77 (56.20%) came from rural (vs. city) and 52 (38%) were the “only-child.” The mean level of frustration tolerance is 13.10 (SD: 6.50); self-esteem, 24.02 (SD: 6.29); hostility, 63.54(SD: 14.23).

### Common method deviation test

Harman's single factor test showed 18 factors with characteristic roots greater than 1. The first factor explained 20.61% of the variance, which was less than the critical value of 40% (45), indicating there was no obvious common method deviation in this study.

### Correlational analysis

Table 1 showed intercorrelations among variables and all correlations were significant (*p *< 0.001). Father absence was negatively correlated with self-esteem and frustration tolerance. Hostility was negatively correlated with self-esteem and frustration tolerance. Both hypothesized mediators, self-esteem, and frustration tolerance, were positively correlated with each other. Since the correlation coefficients between certain variables seem to be quite high (*r* ≥ 0.70), we did the further statistical analysis and precluded multicollinearity between variables (VIF < 10, tolerance values were all greater than 0.4).

**Table 1:** Descriptive statistics and correlation of variables.

	1	2	3	4
1. Father absence				
2. Self-esteem	−0.25***			
3. Frustration tolerance	−0.34***	0.74***		
4. Hostility	0.30***	−0.64***	−0.69***	
M ± SD	3.68 ± 1.13	24.10 ± 6.34	13.12 ± 6.48	63.41 ± 14.26

*** *p* < 0.001.

### Serial mediation analysis model

The correlation analysis results above showed that self-esteem and frustration tolerance met the statistical requirements for mediation testing (46). A serial mediation analysis was carried out using Model 6 in the PROCESS macro for SPSS (44), with father absence as the independent variable, hostility as the dependent variable, self-esteem and frustration tolerance as two mediators, while controlling for gender and age.

**Figure 1:**
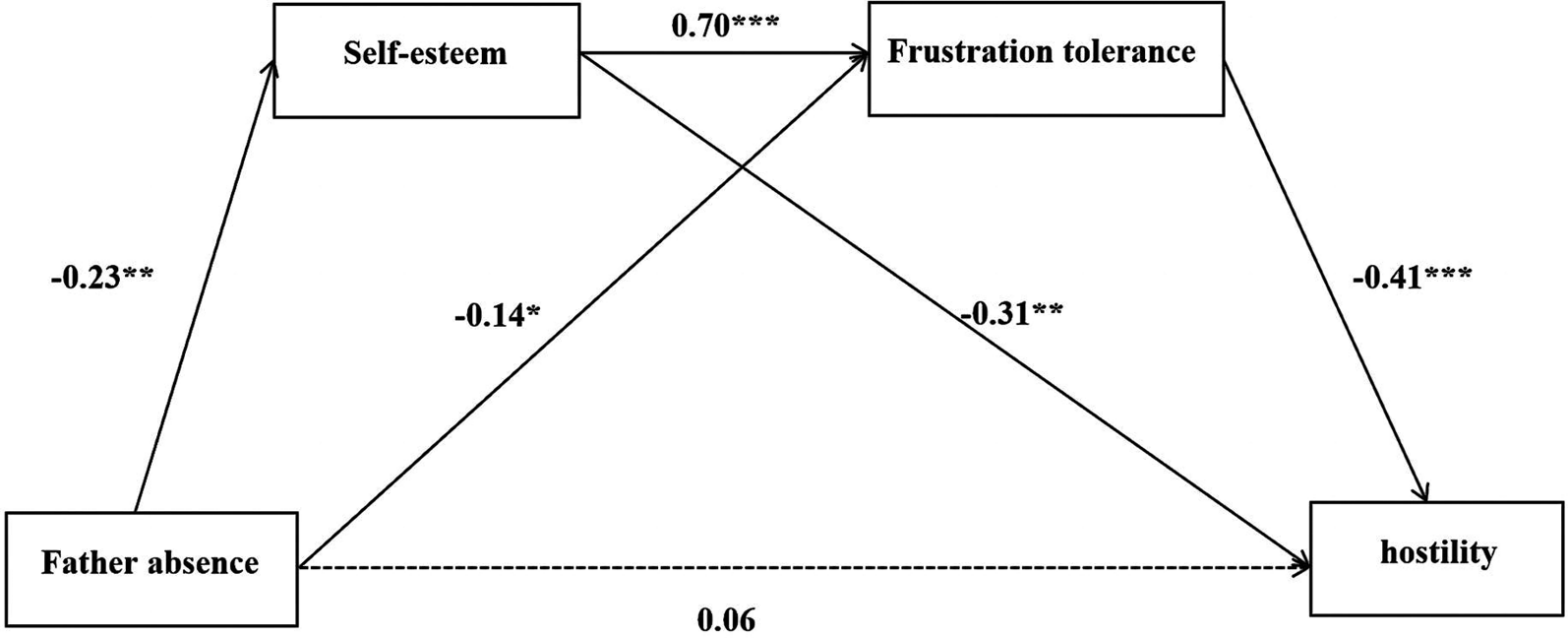
Mediation model between father absence and hostility level.

As shown in Table 2 and Figure 1, the total effect of father absence on hostility was significant (*β *= 0.25, *p *< 0.01). In addition, the direct effect of father absence on hostility was non-significant when mediators (self-esteem and frustration tolerance) were added (*p *> 0.05), indicating a significant full mediation effect. The total indirect effect of father absence on hostility was also significant. Both mediators, self-esteem and frustration tolerance, showed a significant effect on hostility, as represented by corresponding mediator paths. Furthermore, a significant indirect effect was found for father absence through self-esteem and frustration. The indirect effects of father absence on hostility through self-esteem, frustration tolerance, as well as through self-esteem and frustration tolerance serially accounted for 28%, 24%, and 24% of the total effect, respectively. These results showed that father absence did not directly influence hostility, but through low self-esteem and frustration.

**Table 2:** Bootstrap analysis of the mediation effect test.

Mediation Path	Point Estimate	Bootstrap S.E.	BOOSTRAP 5,000 TIMES 95% CI		Effect percentage
			Lower	Upper	
Total indirect effect (ab)	0.19	0.05	0.09	0.29	76%
Ind 1(a1b1)	0.07	0.03	0.02	0.13	28%
Ind 2 (a2b2)	0.06	0.03	0.01	0.11	24%
Ind 3 (a1a3b2)	0.06	0.03	0.02	0.12	24%

Ind 1: Father absence → Self-esteem → Hostility level; Ind 2: Father absence → frustration tolerance → Hostility level; Ind 3: Father absence → Self-esteem → frustration tolerance → Hostility level.

## Discussion

Previous research has shown that high levels of depressive symptoms were strongly associated with high levels of hostility (47). Hostility has been reported to be related to escape-avoidance coping styles and substance use (48). For young people, higher levels of hostility are usually more likely to have interpersonal conflict and rejection. And depressed youths are at higher risk for future hostility than their non-depressed counterparts (15). Therefore, it is necessary to pay attention to the hostility in depressed youths.

The main purpose of this study was to examine the possible roles of self-esteem and frustration tolerance as mediators of the relationship between father absence and hostility in depressed youths. Our results suggested that a lower level of self-esteem, resulting from father absence, was associated with lower levels of frustration tolerance and higher levels of hostility. This indirect path was statistically significant, rendering the direct path from father absence to hostility statistically insignificant. This indicated that the relationship between father absence and hostility was fully, serially mediated by self-esteem and frustration tolerance. To the best of our knowledge, this is the first empirical study in China investigating the relationship between father absence, self-esteem, frustration tolerance, and hostility in youths with major depressive disorder. This study, for the first time, also pointed out the role of self-esteem and frustration tolerance in explaining the link between father absence and hostility.

The positive relationship between father absence and hostility is consistent with previous studies. A growing body of research has shown the negative impacts of father absence on young people, and how the impacts on hostility and delinquency (49–51). Compared to youths with father, the youths with absent father report more hostility. The youths with father absence often suffer chronically from more negative events and pressures (52), and they thus tend to experience more negative affect, which in turn facilitates hostility. In addition, studies have indicated father absence has a negative effect on an individual's social-emotional development, well-being, cognitive ability and mental health and the psychological harm experienced during childhood persists throughout the life course (52).

Central to our research findings was the examination of how self-esteem and frustration tolerance were postulated as jointly mediating variables in the relationship between father absence and hostility in a model. In this regard, we tested three mediation models including two simple mediations and one serial mediation. Our findings demonstrated that the serial multiple mediation effect of self-esteem and frustration tolerance in sequence and the separate mediation effect of them were both statistically significant.

The study indicated that self-esteem was an important mediator in explaining the relationship between father absence and hostility in depressed youths. In a family with a father absence, children receive less encouragement or praise from their fathers, thus resulting in lower levels of self-evaluation. Lower self-evaluation makes it easier to internalize negative evaluations from the outside world, causing lower levels of self-esteem. This process is compatible with Sociometer Theory, illustrating how valued and socially accepted youths are in the eyes of others (53). Young people easily adopt the views that caregivers have about them. Thus, disapproving, unresponsive, and uninterested parents negatively impact self-esteem levels in their children. And low self-esteem will negatively impact an individual's social links, causing the individual to be inconsistent with social norms, thus increasing hostile behaviors (54). Rogers believes that if there is a lack of positive self-attention, individuals are more prone to hostility (55). Boden et al. followed a birth cohort till the age of 25 and found that lower self-esteem at age 15 predicted greater risks of violent offending and higher levels of hostility at ages 18, 21, and 25 (30). In short, a higher level of father absence was associated with a lower level of self-esteem, which was further associated with a higher level of hostility.

Having a high frustration tolerance allows an individual to tolerate setbacks and adopt a positive mental attitude when experiencing adversity, trauma or major negative events (56). Consistent with the existing literature, we found father absence had an indirect effect on hostility through the mediation of frustration tolerance. This finding was compatible with the frustration-hostility theory illustrating that when a person's motivation or behavior is frustrated, it will produce offensive and aggressive reactions (57). Many personality characteristics and willpower qualities are related to how the father brings up the child (21, 22). For instance, depressed youths whose fathers are absent have low levels of frustration tolerance and mental resilience due to a lack of psychological support from their fathers (58). And low frustration tolerance is strongly correlated with high levels of hostility (59). Compared with ordinary teenagers, depressed youths tend to experience more negative events and pressures and a strong sense of frustration, resulting in stronger hostility. High frustration tolerance may be said to act as a positive psychological resource that can buffer against the negative consequences of father absence and reduce the level of hostility.

In addition, a unique finding of the study was that self-esteem and frustration tolerance act as mediators, influencing the father absence-hostility relationship. The results illustrated that self-esteem and frustration tolerance may induce hostility in depressed young people whose fathers are absent. This implied that depressed youths whose fathers are absent have lower self-esteem and tend to adopt negative methods such as self-blame, withdrawal, and fantasies to cope with setbacks. Easily defeated by setbacks, thus, they become hostile. While those with high self-esteem can better regulate their mood and behavior, and thus have good psychological adaptability (60). They can deal with frustration in a more active way and have a higher level of endurance. A high level of self-esteem may improve an individual's frustration tolerance by using some positive coping styles (60).

These findings have important implications for understanding the relationship between father absence and hostility. From the perspective of the serial mediation effect of self-esteem and frustration tolerance on the relationship between father absence and hostility, improving a depressed youth's self-esteem and frustration tolerance will be beneficial for reducing hostility. Therefore, some measures should be taken to enhance self-esteem and frustration tolerance in depressed youths. Regarding self-esteem, for youths who lack parental support, therapeutic interventions (e.g., family therapy) focusing on the interpersonal relationship between the individuals and their parents may be helpful (61). In terms of frustration tolerance, psychological education programs could be carried out to enhance positive self-efficacy and psychological problem-solving competencies. In addition, a study showed that painting group psychological counseling to improve self-esteem was an effective method to enhance frustration tolerance (62). Overall, our findings provide a new perspective for reducing the hostility degree of depressed youths. In the future, researchers and practitioners should further investigate the approaches of lowing the depressed youth's hostility by enhancing self-esteem and frustration tolerance.

## Limitations

Several limitations should be acknowledged when interpreting the findings in the study. First, the cross-sectional design of the study may preclude any causal relationships among the variables to be drawn. However, our serial mediation model is built upon robust evidence from past longitudinal studies, and we believe our model still provides useful information and suggests causal relationships, which warrant future longitudinal studies for further validation. Second, we tested the model in a clinical sample of depressed youths in Wuhan Mental Health Center, which may not be representative of depressed youths in other areas, and moreover, non-depressed youths. In addition, the participants were all inpatients with major depressive disorder, ignoring those in outpatient department, which could result in potential selection bias. Further research is needed to determine whether the model is applicable to outpatients with major depressive disorder. In conclusion, future studies may consider testing this model in depressed youths in outpatient department and other areas, as well as young people in the general community. Third, we did not assess other potential unobserved confounders, such as the severity of depressive symptoms and the use of antidepressant drugs, which should be assessed and controlled for in future studies to test the model more robustly. Forth, Major Depressive Disorder (MDD) is a heterogeneous disease and treating this group as a single group may mask differences between sub-groups of MDD patients. Despite these limitations, our study serves as a first attempt to test a serial mediation model of father absence and hostility mediated by self-esteem and frustration tolerance among depressed youths and provides useful information and guidance for future studies.

## Conclusions

Our study tested a serial mediation model of self-esteem and frustration tolerance as mediators between father absence and hostility among depressed youths. Our results suggested that high levels of father absence had an adverse effect on levels of self-esteem, which decreased levels of frustration tolerance, and thus higher levels of hostility among depressed youths. These findings provided implications for future intervention programs to focus on improving self-esteem and frustration tolerance among depressed youths with father absence to reduce their hostility levels.

## Data Availability

The raw data supporting the conclusions of this article will be made available by the authors, without undue reservation.
